# Approaches to Medical Decision-Making Based on Big Clinical Data

**DOI:** 10.1155/2018/3917659

**Published:** 2018-06-04

**Authors:** V. L. Malykh, S. V. Rudetskiy

**Affiliations:** Medical Informatics Research Center, Ailamazyan Program Systems Institute of RAS, Pereslavl-Zalessky, Russia

## Abstract

The paper discusses different approaches to building a medical decision support system based on big data. The authors sought to abstain from any data reduction and apply universal teaching and big data processing methods independent of disease classification standards. The paper assesses and compares the accuracy of recommendations among three options: case-based reasoning, simple single-layer neural network, and probabilistic neural network. Further, the paper substantiates the assumption regarding the most efficient approach to solving the specified problem.

## 1. Introduction

Providing support to medical decision-making is one of the most urgent issues in healthcare automation. It has been repeatedly noted in different articles, reports, and forum discussions [1] both in Russia and abroad that MIS introduction requires a considerable extra effort from users—doctors in the first place—to enter primary data into the system. Naturally, doctors expect practical intelligent outcomes from big clinical data accumulated by modern MISs. Handler et al. [2] present the operating paradigm of 5th generation MISs, referred to as “MIS as Mentor.” Malykh et al. [3] adds one more qualitative characteristic to the above paradigm—“MIS as automated mentor.” “It is advisable to abandon the practice of active user dialogs typical of expert systems, involving requests for data that the system considers missing from the user, and substitute the dialog with an automated nonintrusive algorithm that draws its own logical conclusions and generates recommendations in a completely automated manner based on available data, without involving the user in the process. The user may either accept or ignore the system's prompts and recommendations; however, they will not provoke rejection in users if delivered automatically without requiring a dialog with the system.” To provide a brief qualitative description of this increasing subjectivity of MISs, we have proposed a new term “active MIS” that emphasizes a certain degree of independence from users or subjectivity of the cyber system. Kohane [4] presents the most “balanced” definition of personalized medicine, “personalized medicine is the practice of clinical decision-making such that the decisions made maximize the outcomes that the patient most cares about and minimize those that the patient fears the most, on the basis of as much knowledge about the individual's state as is available.” This perception of personal medicine is focused on clinical decision-making and once again exhibits the urgency and importance of scientific research in the area. Therefore, building an automated active mentor-type system that provides recommendations regarding treatment and diagnostic activities to the doctor is an urgent practical task.

Butko and Olshansky [5] and Kotov [6] provide a retrospective overview of approaches to building medical decision support systems. The applied approaches were restricted in many respects by the abilities of computers at that time. Accordingly, there was no such problem as processing big medical data. Technologies have evolved to the point when big medical data (both on individuals and the population in general) collection and accumulation is finally feasible. At the same time, big data processing and intelligent system learning methods were evolving as well. Along with “deep learning,” the term “deep patient” [7] was coined, meaning the opportunity to extract increasingly more complete, deep, and valuable information about patients from big clinical data using deep learning methods.

Malykh et al. [8] mention the possibility of creating national-scale clinical data banks. Herrett et al. [9] provide an example of a database (DB) containing anonymous medical records on primary healthcare services provided. This DB was created by a joint effort of 674 general practitioners and covers over 11.3 mn patients in Great Britain.

Decision-making in hospitals has evolved from being opinion-based to being based on sound scientific evidence. This decision-making is recognized as evidence-based practice. Perpetual publication of new evidence combined with the demands of everyday practice makes it difficult for health professionals to keep up to date [10].

A large number of publications are devoted to medical decision support systems (DSSs), including publications in specialized scientific journals (*Artificial Intelligence in Medicine, BMC Medical Informatics and Decision Making, International Journal of Medical Informatics, Medical Decision Making*, etc.). The work does not aim to give an overview of different approaches to making of decision support systems, referring readers to the original reviews [11–13]. We can give a few definitions for decision support system from Wikipedia: “Clinical Decision Support systems link health observations with health knowledge to influence health choices by clinicians for improved health care” and “active knowledge systems, which use two or more items of patient data to generate case-specific advice.” No one doubts the feasibility of such systems and that they have a positive impact on professional practice, patient outcomes, length of hospital stay, and hospital costs. The main problem is to find effective approaches to building such systems.

A number of contemporary approaches to medical decision support system development are listed by Malykh et al. [14].

The first one of these approaches involves provision of relevant data sources to doctors, helping them make decisions independently. The system does not recommend any final solutions—instead, it suggests data sources to study and find answers to current questions (Evidence-Based Clinical Decision Support at the Point of Care | UpToDate URL: http://www.uptodate.com/home).

The second approach is to use clinical pathways. Clinical pathways represent prescriptive models of the standard healthcare procedures that need to be undertaken for a specific patient population. Instances of the clinical pathways (also known as cases) describe the actual diagnostic-therapeutic cycle of an individual patient [15]. But even in the case of the use of clinical pathways, the process of clinical decision-making has high complexity. While the medical knowledge used in the decision process comes partially from published research contributions and widespread medical guidelines (with various kinds of evidence levels), it is generally accepted that the decision process is profoundly influenced by the expertise and experiences of the involved medical experts [15].

The third approach involves development of a large number of individual narrow-focused decision support systems. This approach helps achieve top quality when solving isolated problems [6, 12]; however, it is almost impossible to apply it to big clinical data.

The fourth approach that claims to have a global scope of application is focused on building a cognitive system capable of self-learning and knowledge digestion directly from nonformalized text sources (IBM Watson http://www.ibm.com/smarterplanet/us/en/ibmwatson/).

None of the reviewed approaches is immaculate. All of them require efforts of experts and regular updates of knowledge bases. Moreover, each of the approaches is in fact tailored to specific purposes.

The latest Russian-language review [12] noted that clinical decision support systems have not become widespread in Russia. This is due to the complexity of the development of such systems, the specific character of the systems already developed, and the need to involve high-class experts in the development.

In this paper, we will review general approaches to decision support system development based on nonreduced big clinical data. The main expectations related to application of general approaches ensue from the case-based nature of decision-making in healthcare, and the assumption that big clinical data already contain enough knowledge for efficient decision-making.

There are two other factors that draw attention to systems based on machine learning or precedent approach.

First of them is that there are trends in the development of our civilization which include an explosive development of information technologies (among them M2M, Big Data, and IoT), their strong need for formalized knowledge, and practical absence of qualified experts who could formalize that knowledge. The chief editor of the Rational Enterprise Management (REM) magazine (Russia) holds regular discussions on a wide range of problems including the above-mentioned ones. Results of the discussions are published in the REM editor's column. The guests of a recent discussion [16] included Igor Rudym (Intel), Dmitriy Tameev (PTC), Alexander Belotserkovskiy (Microsoft), Igor Girkin (Cisco), and Igor Kulinitchev (IBM). All the participants agreed that, nowadays, the key challenge of IT development is not associated with hardware or software, but it needs breakthrough approaches to data analysis.

As for the second factor, it is obvious that, nowadays, there are no qualified experts in the field of knowledge even in key branches. The actual situation is even more critical as the experts who are able to solve at least a part of these problems are not able to cope with ever increasing information flow. From this point of view, precedent-based DSSs practically need no experts. Experts may be needed for enhancing or optimizing existing medical data bases and knowledge bases [14].

## 2. Model and Methods

We regard the diagnostic and treatment process (DTP) as a discrete controlled process with a memory. The model was first introduced by Malykh et al. [17, 18] written in Russian. In English, the model is described by Malykh et al. [8, 19]. To ensure further understanding of the essence of the problem, let us provide an extract from the source.

Modern medical information systems store electronic medical records and contain descriptions of millions of various clinical cases. The degree of formalization of clinical data stored in MISs varies. MISs model the diagnostic and treatment process as a sequence of controlling events reflecting diagnostic and treatment activities, and a sequence of monitoring events describing the condition of the patient. Controlling events are well formalized; medical organizations keep statistical and business records of such events, plan them, and allocate required resources. Medical data related to monitoring of patients' condition are less formalized and may be partly available in the form of plain text medical documents.

Previous studies provide evidence that is possible to model the DTP using controlled stochastic Markov processes [18]. The model is based on the assumption that the DTP is a discrete controlled process. The model introduces the notions of control U and state X. Controls are diagnostic and treatment decisions made and executed in future. Controls are different diagnostic and treatment activities prescribed by doctors, including diagnostic tests, medicines, surgical interventions, various procedures, and manipulations. The choice of diagnostic and treatment activities is based on the accumulated medical knowledge and the doctor's individual experience. The scope of potential diagnostic and treatment activities comprises previously applied measures with proven efficiency. Controls are essentially precedent dependent.

The choice of control (X_*i*_, U_*i*_) depends not only on the current state (X_*i*_) but also on the overall background of the process as well as controls applied at earlier DTP stages {*i*, *i* − 1, *i* − 2,…}. This is due to the specific features and nature of the treatment process. To take the process memory effect into account, it is proposed to include the integral property of the relevant control in the extended state of the discrete process. Each control in the DTP can be associated with some integral property of such control. For example, such integral properties include full dose of medicine taken by the patient at this stage of the DTP or full dose of radiation the patient was exposed to in the course of radiotherapy. The frequency (number) of application of different control elements is also regarded as an integral property (e.g., the number of assigned ECGs).

DTP modeling based on the Markov process appears sufficiently substantiated [17, 18, 20], especially in cases involving DTP description for inpatients with strictly regular monitoring and medical decision-making.

Thus, in the model, the DTP is represented by a sequence of vectors of equal length and structure V split into two components—control U and monitored properties X. Control components have non-negative numerical values. A zero value of control at this stage of the process means that this kind of control has never been applied before, starting from the beginning of the process and up until this step inclusively. Components of monitored properties are of different nature. They can be dimensional physical values or non-numerical, for example, assignment of a property's value to a specific class. Since it is almost impossible to monitor all the properties at the same time, certain components of properties may be unknown to us. When applying different methods to the model, we may need to digitize non-numerical values of components and identify missing values of monitored properties.

### 2.1. Definition of the Objective

**Figure 1:**
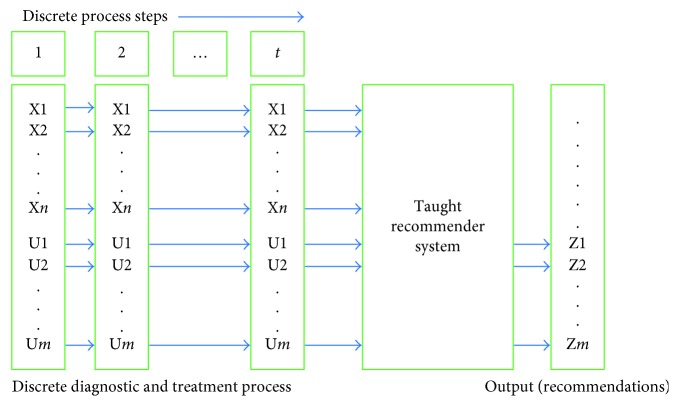
Recommender system.

We will review several methods that can be applied to build a cybernetic taught system. The input into the system will be a sequence of vectors describing a discrete DTP in accordance with the presented model. The output will consist of recommendations proposing diagnostic and treatment options (choice of controls) for this particular state of the process. A diagram of the system is presented in Figure 1.

Let us define the objective more accurately and assume that each DTP model is considered in the context of an already available predominant diagnosis. For each model, we have an array of earlier observed DTP implementations. Such implementations are sources of knowledge about treatment of a particular nosology, and they are used to teach a cybernetic recommender system to operate in the given context. Based on available DTP implementations, we defined a glossary of controls and monitored properties for each model. Issues related to normalization of primary data, outlier testing and exclusion, and approaches to data generalization based on assignment of monitored properties to generic classes are beyond the scope of this paper [18]. It might also be necessary to extract data directly from the text of medical documents. Once this enormous and useful effort is completed, we will have a bank of clinical data containing sets of DTPs with homogeneous descriptions for each nosology present in the bank. We would like to emphasize that no primary data reduction is envisaged, such as focusing solely on properties meaningful in the context of the relevant nosology. Data are extracted from the MIS “as is”—exactly as there were entered in the MIS by doctors, assuming such data will most likely contain significant and meaningful information for the relevant nosology.

Finally, let us provide examples of typical properties of nonreduced primary data. We believe that a process ensemble in a data bank may reach 10^3–10^6 processes for an individual nosology. The dimension of a vector describing one step of a discrete DTP exceeds 10^3. The dimension of a control (output of the cybernetic system) may also exceed 10^3.

The case-based approach, including its application to medical decision support, has been described in sufficient detail in multiple sources [6, 14, 19]. The main idea of the case-based approach is quite simple—find a clinical case in the DB similar to the one in focus and use it for medical decision support purposes. Additionally, clinical cases used as precedents during the search can be filtered taking into account such factors as reputation of medical organizations that such cases originate from, reputation of doctors who created such cases, or relevance of the cases in view of contemporary medical technologies. To ensure successful application of the case-based approach, it is necessary to have representative DBs of clinical cases.

**Figure 2:**
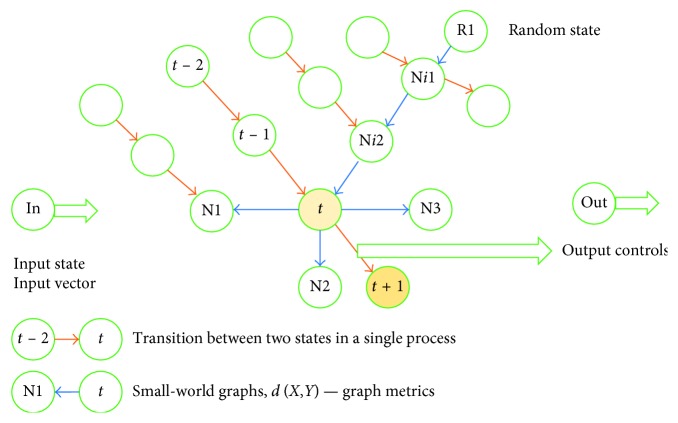
Structure of a case-based system.

Malykh et al. [14] present assessment results with respect to the accuracy of diagnostic and treatment activities recommended using case-based reasoning. The structure of the cybernetic system chosen for the approach in focus is presented in Figure 2.

We have a network and each node in it is presented by a single DTP state. Each individual DTP represents a specific route within the network (routes are marked in Figure 2 by orange arrows). In the model, each state is represented by vector V. A metric or distance *d*(*X*, *Y*) is defined for each state. Based on the defined metric or distance, a small-world graph is plotted [21]. For each node in the small-world graph, *n* (graph parameter) closest neighbors are identified. In Figure 2, closest neighbors are marked with pointing blue arrows; four closest neighbors are specified for node *t*—N1, N2, N3, and N*i*2.

Here is how the recommender system operates. The input into the system is a current state of the DTP: The situation when the input contains the entire implemented sequence of process states is beyond the scope of this paper. Several nodes are randomly selected on the small-world graph (R1 in the example presented in Figure 2). From original nodes towards their closest neighbors, we go down to the graph node minimizing locally the distance between the node (R1 → N*i*1 → N*i*2 → *t* in Figure 2) and the input state. The best of all the identified local minimums is selected. It will be regarded as the closest neighbor of input state In. At this point, the recommended control can be calculated as the difference between integral properties of control components of two vectors. In Figure 2, these are state vectors (*t* + 1) and (*t*). The recommended control is U = U(*t* + 1) − U(*t*).

It is easy to assess the scale of the network in focus. In the example with 1,000 processes for one main nosology with the average duration of the process equal to ten days, we will need 10,000 network nodes. Each node will store a vector with the dimension 1,000 or higher. Computational experiments show that 0.5–1% of the total number of nodes is sufficient as random initial network nodes. In case with 10,000 nodes, the number of initial nodes will be 50–100. The descent along the small-world graph was quick, and the routes did not exceed 10 steps on average. The number of edges originating from each node in the small-world graph was equal to 8. The top-down assessment of the number of metric calculations in this case equals to 100∗10∗8. It is possible to accelerate the calculations by splitting the small-world graph into layers corresponding to specific DTP lengths and searching for closest neighbors within the layer corresponding to the input state. In the above example, we would have layers consisting of 1,000 states, and we would search for closest neighbors starting from 5 to 10 randomly selected nodes. This is fully acceptable in view of the computational requirements: computational experiments show that, in this case, computations can be performed almost real-time.

Let us review the network teaching process. Teaching means adding new DTP implementations to the network. The number of metric calculations *d* when adding *k* states of a new process to the network containing *m* states equals to *k*∗*m*. This is absolutely acceptable in view of the computational requirements As a result, new knowledge will be added to the network, and it will be extended by *k* new nodes and (*k* − 1 + *k*∗*n*) edges. It is essential to emphasize the network's sensitivity to new knowledge. Apparently, any newly added DTP implementation may have a significant impact on the decision recommended by the system if the closest neighbor is selected from the added implementation. It may be asserted that the network digests new knowledge and starts applying it immediately. We will not see this in approaches described below.

**Figure 3:**
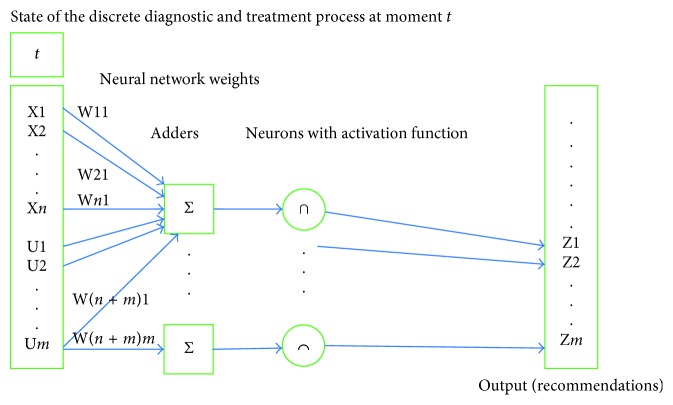
Neural network.

As an alternative approach, let us consider a basic neural network with a single layer. The structure of the network is outlined in Figure 3.

Current DTP state is used as input to a basic one-layer neural network. The network contains *m* adders and *m* neurons in accordance with the dimension of control component U. In the output, each neuron has either one of the values {0,1}. Output 1 of neuron *i* means the system recommends control U_*i*_ for this state. Output 0 of neuron *i* means the system refuses to recommend control U_*i*_ for this state.

Let us refer to the network scale as an example. Let the dimension of input vector be 1,000 and that of the control component 500. In such case the teaching process will involve definition of 1,000∗500 weights. Let us remark that no major reduction of the neural network is possible to solve the above problem. The reason is that the dimension of the control component is the number of diagnostic and treatment activities that can be prescribed for this nosology, including coexisting illnesses. And this number is enormous. Adding new layers to the neural network will only make matters worse by increasing the number of taught parameters.

Let us examine the network teaching process. Initially, a certain set of DTPs is selected and used for network teaching purposes, including calculation of weights. New DTP implementations emerge. How should we use this new knowledge? If a sufficiently large volume of DTP implementations was used to teach the network (1,000 to 10,000) and new implementations constitute an insignificant share of the teaching sample (e.g., 100 new implementations versus 10,000 is merely 1%), it can be asserted that network re-teaching will not result in any noticeable changes in teaching parameters, and consequently, any major variations in the network's output. This kind of network is rough and conservative; it can “digest” new knowledge only when the volume of such is sufficient. In this respect, neural networks are not as good as networks applying the case-based approach.

**Figure 4:**
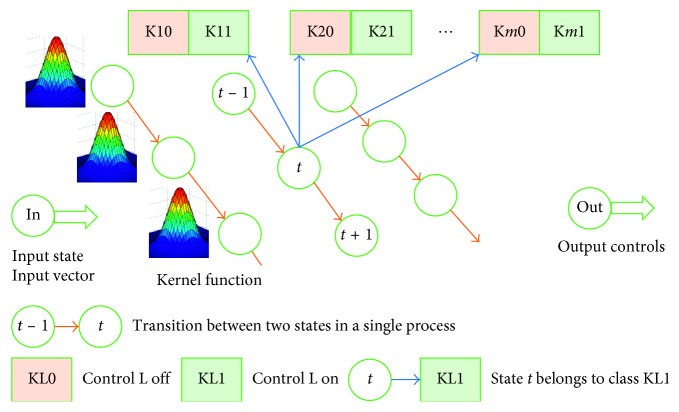
Probabilistic neural network.

As another alternative approach, let us consider a probabilistic neural network. The structure of the network is outlined in Figure 4. For each state (state vector V), there is one kernel function f(V) common for all the states. In our case, we used a multivariate Gaussian distribution function with a diagonal covariance matrix. The kernel function includes parameter *σ* affecting the function's width. Each state is classified into 2*m* classes, where *m* is the dimension of the control component. If a doctor applies control L to state *t*, then *t* belongs to class KL1; otherwise, it belongs to class KL0.

**Figure 5:**
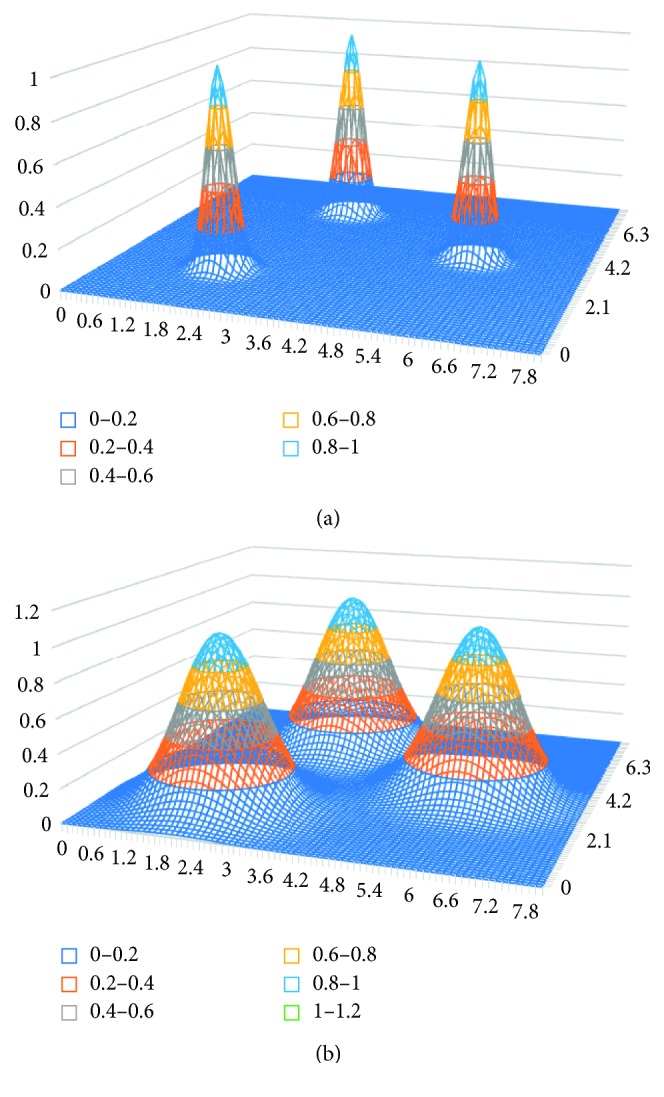
Impact of control parameter *σ* on kernel functions and type of distribution.

Figure 5 shows the impact of control parameter *σ* on the type of distribution.

Now, a probability density function can be “restored” for each class. For input vector In, we apply Bayes' formula to calculate the posterior probability of belonging to each class and generate recommendations regarding the choice of diagnostic and treatment activities for this state.

Let us refer to the network scale as an example. Let the dimension of the input vector be 1,000, the dimension of the control component be 500, and the teaching sample contain 1,000 processes with 10 states in each. We will need to calculate 10,000 kernel functions and then calculate 1,000 posterior probabilities of the input vector belonging to each class for various distributions of kernel function supports for 500∗2 different classes.

Let us examine the network teaching process. The teaching process is focused on adding new DTP implementations to the network, including assignment of states to different classes. If the number of new implementations is a small share of the teaching sample used earlier, it can be asserted that adding new implementations will have no major impact on the network's output. The probabilistic neural network proves to be rough and conservative; it can “digest” new knowledge only when the volume of such is sufficient. In this respect, probabilistic neural networks are not as good as networks applying the case-based approach.

## 3. Results

We performed computational experiments for a network built using the case-based approach in 2015-2016. The results were published in Malykh et al. [14]. To compare different approaches to the problem, we will present the results of paper [14] in a slightly modified format.

Table 1 shows that the number of correct recommendations (TP True Positive) varies from 58.7 to 94.9% depending on the type of nosology. The majority of recommendations match the doctor's actions.

In the matter of neural networks, computational experiments for all nosologies listed in Table 1 required quite a lot of time and computing power. The practical value of such full-scale experiments was unclear. Therefore, it was decided to limit computational experiments to estimations for nosology J13.  Table 2 contains general information about the experiment with a single-layer neural network.

Let us emphasize that the volume of statistics on this illness stored in the DB has increased compared to an earlier experiment involving the same nosology—from 166 to 266 completed clinical processes. Controls included all types of drug prescriptions (222 different pharmaceutical products in our case). Data normalization involved adjustment of prescribed dosages of pharmaceutical products to unified dose units. The only monitored variable was “inpatient days.” Inputs also included bias. 49,728 weights had to be determined. The optimized target function was a quadratic residual between neural network output and control components monitored in control samples, adjusted to (0, 1). We used a nonstandard neurons activation bell curve (Gaussian function). This choice of activation function was based on the fact that integral values of many controls had apparent limits stipulated by Russian federal healthcare standards (standards of the Russian Ministry of Health). Different insurance programs also limit integral values of controls. Healthcare providers will not exceed these limits unless they find it necessary. Formally, with respect to the model, it means that once an integral property of a control reaches a certain limit, it stops growing further or such growth is highly unlikely. The gradient of the target function with respect to weights was calculated explicitly, and the steepest descent method was applied. Teaching included 1,006 descent steps. Criteria reflecting the accuracy of the neural network are presented in Table 3.

**Figure 6:**
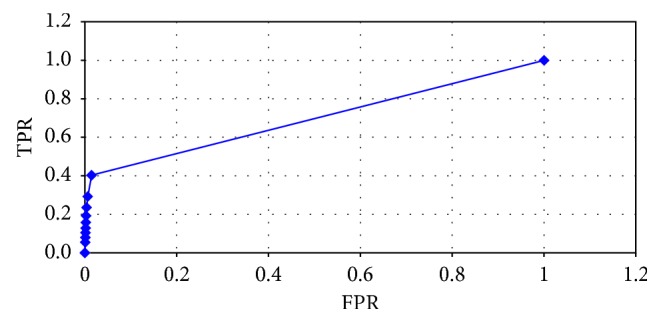
ROC error curve.

The relevant receiver operating characteristic (ROC) error curve is shown in Figure 6.

Results of the experiment based on a probabilistic neural network are presented in Table 4. The state vector dimension was equal to 639. The control component included 125 diagnostic tests, 200 laboratory tests (different kinds), 222 different pharmaceuticals, 87 medical treatments, and 4 controls classified as “others.” The only monitored property was “inpatient days.” The number of kernels (states) in the teaching sample of 266 processes was 4,361. The dimension of the state vector in the probabilistic neural network was almost three times the dimension of the state vector in the single-layer neural network (639 versus 223). To make the results of both networks comparable, the output of the probabilistic neural network was considered to be the same as for the first neural network. The output was a vector with a dimension of 222, related to prescription of different pharmaceuticals. Both neural networks generated 36,408 positive and negative recommendations for the control sample. The experiment involved one control parameter *σ*, a multiplier for a diagonal covariance matrix used in the kernel function (multivariate Gaussian distribution of independent random variables). A value grid was predetermined for the parameter *σ*, and the best value of the parameter was chosen based on experimental calculation results [22]. Calculations were performed for the following values of *σ*: (0.1, 0.5, 1, and 2.5). The best results were obtained for *σ* = 2.5. They are presented in Table 4. Let us emphasize that standard deviation values of the state vector components calculated for the teaching sample were significant and often exceeded average values. The multiplier equal to 2.5 yields “wide” kernel functions (see the rightmost distribution in Figure 5). With “sharp” kernel functions (*σ* = 0.1), the results were obviously worse.

## 4. Summary

The focus of this paper was how to build a medical decision support system based on big clinical data. The authors review general approaches to the problem that do not involve individual models for specific nosologies and neither do they require engagement of experts in the relevant subject area to such modeling or knowledge extraction from data. Data are extracted from the MIS without reduction, “as is.” It is assumed that the data contain significant information reflecting medical knowledge and contemporary medical treatment technologies. Three different approaches to big clinical data processing were examined: (1) case-based reasoning for decision-making; (2) decision-making based on a single-layer neural network; and (3) decision-making based on a probabilistic neural network. Experimental calculations were performed to assess the accuracy of recommendations generated using different approaches.

Drawbacks of the above neural networks with respect to the given problem were identified. The overall accuracy of provided recommendations was rather high. Moreover, the accuracy of negative recommendations that the neural networks learned to provide was very high (98–99%). However, the accuracy of positive recommendations provided by the neural networks was not so high (40–55%, which is obviously insufficient for successful practical application). Another disadvantage of neural networks is their rough and conservative nature, particularly when digesting isolated portions of new data with the volume insignificant compared to previously available data.

The case-based approach to decision-making yielded more accurate recommendations (59–95%), which is sufficient for its successful practical application. Another advantage of the case-based approach is its sensitivity to new data. With respect to calculations, the case-based approach is also more efficient compared to other options under consideration as it ensures a high operating speed of the decision support system, thus making it acceptable for practical application. These are the key findings of the study conducted.

This offers encouraging prospects for designing and developing decision support systems for physicians based on empirical components of medical knowledge. This approach also corresponds to existing case-based character of management and decision-making in medical practice. So far, the results indicate that precedent-based approach has a high effectiveness and could naturally enhance other approaches to supporting physicians' decision-making, particularly knowledge-based ones. The obvious practical value of this approach lies in the fact that it can be complementary to other knowledge-based approaches (clinical pathways, Evidence-Based Clinical Decision Support, expert systems, Watson, etc.). The doctor will be able to make decisions based on the best examples of medical practice, finding precedents of clinical cases close to the given case.

The constraints of precedent-based approach include the need for a representative database of verified precedents excluding medical errors. From another perspective, precedents with corrected errors are of particular interest to physicians training and further prevention of such errors. The information about the results of these errors and possible ways of correcting them is also valuable. Thus, precedent-based approach could be widely spread as an educational tool. On the other hand, the precedent-based approach does not imply formalization of medical knowledge, which entails poor cognitive justification of generated recommendations. Consequently, justifications only describe how other patients were treated in similar clinical cases. There are also problems with optimization of provided metrics, compression of state descriptions, and construction of training procedures. These problems are connected with high dimensionality of the space of state characteristics and samples of clinical precedents. However, discussion of these issues and possible ways of addressing them has been left outside of this research [14].

In further studies, we are going to focus on detailed application of the case-based approach, analyze metrics, and distances not only for pairs of vectors but also for pairs of vector sequences, and examine issues concerned with intelligent normalization of primary data and data extraction from plain texts of medical documents.

## Figures and Tables

**Table 1:** Accuracy assessment of recommended diagnostic and treatment activities for seven nosologies using the case-based approach.

MKB-10 code/nosology	*Total number of clinical precedents/number of control precedents*	Number of correct recommendations among control precedents	Number of recommendations with a different control level among control precedents	Number of diagnostic and treatment activities the decision support system was unable to provide recommendations for among control precedents
	Number of states/number of controlled variables	Absolute value/share in the total number of diagnostic and treatment activities	Absolute value/share in the total number of diagnostic and treatment activities	Absolute value/share in the total number of diagnostic and treatment activities
J13/pneumonia due to *Streptococcus pneumoniae*	*166/11*	6788/81.6%	3923/47.2%	1530/18.4%
	2938/118			

K80.1/calculus of gallbladder with other cholecystitis	*1018/128*	34468/76.7%	18390/40.9%	10490/23.3%
	12853/931			

H25.1/age-related nuclear cataract	*1205/121*	3522/94.9%	539/14.5%	189/5.1%
	5509/293%			

H26.2/complicated cataract	*1255/126*	4362/91.4%	1617/33.9%	408/8.6%
	5778/249%			

I67.4/hypertensive encephalopathy	*1336/134*	65678/72.4%	37563/41.4%	25060/27.6%
	23165/1431			

I67.9/cerebrovascular disease, unspecified	*1403/141*	58649/75.4%	32447/41.7%	19117/24.6%
	24875/1518			

N20.1/calculus of ureter	*1632/164*	17489/58.7%	9948/58.7%	12291/41.3%
	15922/205			

**Table 2:** Accuracy assessment of recommended diagnostic and treatment activities for nosology J13 based on a single-layer neural network.

MKB-10 code/nosology	*Total number of clinical precedents/number of control precedents*	Number of correct positive recommendations among control precedents	Number of incorrect positive recommendations among control precedents	*Total number of negative recommendations/total number of positive recommendations*
				Share of correct negative recommendations/share of correct positive recommendations
	Number of neural network inputs/number of neural network outputs (number of controlled variables)	Absolute value/share in the total number of positive recommendations	Absolute value/share in the total number of positive recommendations	Absolute value/percent
J13/pneumonia due to *Streptococcus pneumoniae*	*266/11*	339/40.31%	502/59.69%	*35567/841*
	224/222			98.55%/40.31%

**Table 3:** Accuracy of recommended diagnostic and treatment activities for nosology J13 based on a single-layer neural network with an activation threshold equal to 0.1.

Absolute values (neuron activation threshold equal to 0.1)			
TP	339	502	FP
TN	35,052	515	FN

Percent (neuron activation threshold equal to 0.1)			
TP	40.31%	59.69%	FP
TN	98.55%	1.45%	FN

TP, true positive; FP, false positive; TN, true negative; FN, false negative.

**Table 4:** Accuracy of recommended diagnostic and treatment activities for nosology J13 based on a probabilistic neural network with *σ* = 2.5.

Absolute values (*σ* = 2.5)			
TP	233	191	FP
TN	35,376	608	FN

Percent (*σ* = 2.5)			
TP	55.0%	45.0	FP
TN	98.31%	1.69%	FN
